# Efficacy of Mandala Coloring Intervention on Executive Functioning and Emotional & Motivational Self-Regulation Among Children With Symptoms of Attention Deficit Hyperactivity Disorder

**DOI:** 10.7759/cureus.46919

**Published:** 2023-10-12

**Authors:** Chhavi Singh, Surekha C, Jaishree K

**Affiliations:** 1 Psychology, Christ (Deemed to be University), Ghaziabad, IND; 2 Rehabilitation, Aashritha - School for Differently Abled, Hyderabad, IND

**Keywords:** emsrq, executive functioning, adhd symptoms, mandala coloring, adhd

## Abstract

Introduction: Attention deficit hyperactivity disorder (ADHD) is the most common neuropsychiatric condition of childhood. There is a sizable subset of children with ADHD symptoms in whom early intervention can prevent their progression into fulminant ADHD. However, the therapeutic options for ADHD symptoms are limited. Mandala coloring is a form of art therapy that may be used in these children, but there is a dearth of literature regarding its efficacy.

Method: This is a prospective cohort study on 120 children aged six to 10 years diagnosed with ADHD symptoms. The children were divided into two equal groups of intervention and control. Children in the intervention group were given mandala coloring intervention for 10 weeks duration. Pre-test and post-test values of executive functioning and emotional and motivational self-regulation (EMSRQ) were compared between the two groups by using SPSS 23 (IBM Corp., Armonk, NY).

Results: The demographic data and baseline characteristics were comparable in both groups. Post-test values showed significant improvement across all parameters of executive functioning in the intervention group. However, the difference was not significant in EMSRQ. At follow-up, parents reported improvement in academic performance, concentration, and mindfully focusing on a single activity for a longer duration of time.

Conclusion: The attention span and cognitive abilities of children at risk for ADHD may be improved with the relatively simple yet effective intervention technique of mandala coloring that can easily be administered by parents at home. Further research is needed to compare its efficacy with other treatment modalities.

## Introduction

Mandala coloring is a form of therapeutic art involving coloring intricate and symmetrical patterns. These are geometric designs that start from a central point and radiate outward, forming a circular or symmetrical pattern [1]. A symmetrical mandala can both be drawn or colored to enhance positive effects on cognitive function and emotional well-being by reducing the "inner chaos" of people's emotional states [2]. Coloring mandalas can promote relaxation, mindfulness, and creativity, aiding in relaxation, emotional and creative expression, focus, and self-regulation in children, thereby benefiting their overall cognitive functioning. Based on the available literature, mandala coloring and drawing have been found to be useful in people with dissociative disorders, attention deficit hyperactivity disorder (ADHD), and dementia [3-5].

Art therapy, such as mandala coloring, is effective because of the unique nature of the art-making process [6]. The process of art-making involves an interplay of motor function, attention, and impulsivity, which are reflected in the line quality, choice of materials, degree of organization, and completeness of the artwork.

The brain neuronal networks involved in attention and focus may benefit from the strengthening gained through prolonged concentration of work during mandala coloring. Working memory, cognitive flexibility, attention span, and interference control are part of executive functioning abilities that lead to the effective completion of the task. According to Curry and Kasser [7], healthy adults who color mandalas can increase their attention span. These results collectively imply that mandala coloring can enhance executive functioning abilities and facilitate more successful task completion by enhancing attentional control, working memory, cognitive flexibility, and problem-solving skills. According to Babouchkina and Robbins' study [8], adults who color mandalas, circular and symmetrical figures that draw the eye to their centers, experience fewer negative mood symptoms. These researchers hypothesized that the circular shape of mandalas is an "active ingredient" in this process.

The majority of the studies on mandala coloring are done on adults or children with other disorders [7,8]. Only a few studies have looked at the effect of mandala coloring in children with ADHD symptoms, and there are no studies on the Indian population even though the concept of mandala originated in the Indian subcontinent. The purpose of this study is to ascertain how executive functioning of children with ADHD symptoms is affected by mandala coloring.

## Materials and methods

With institutional review board approval, a prospective cohort study was performed at a school to compare the effect of mandala coloring on executive functioning and emotional and motivational self-regulation (EMSRQ) of children with ADHD symptoms in intervention and control groups.

Participant selection

The screening test was conducted between January 2022 and June 2022 for 250 children aged six to 10 years for diagnosis of ADHD symptoms. Children with average intelligence and between 84 and 94 percentile on the ADHD-Symptoms Rating Scale (ADHD-SRS) scale [9] were randomly selected for the study. Children with any other disability, those going through other therapy modalities, and those whose parents were suffering from mental health conditions were excluded from the study.

**Figure 1:**
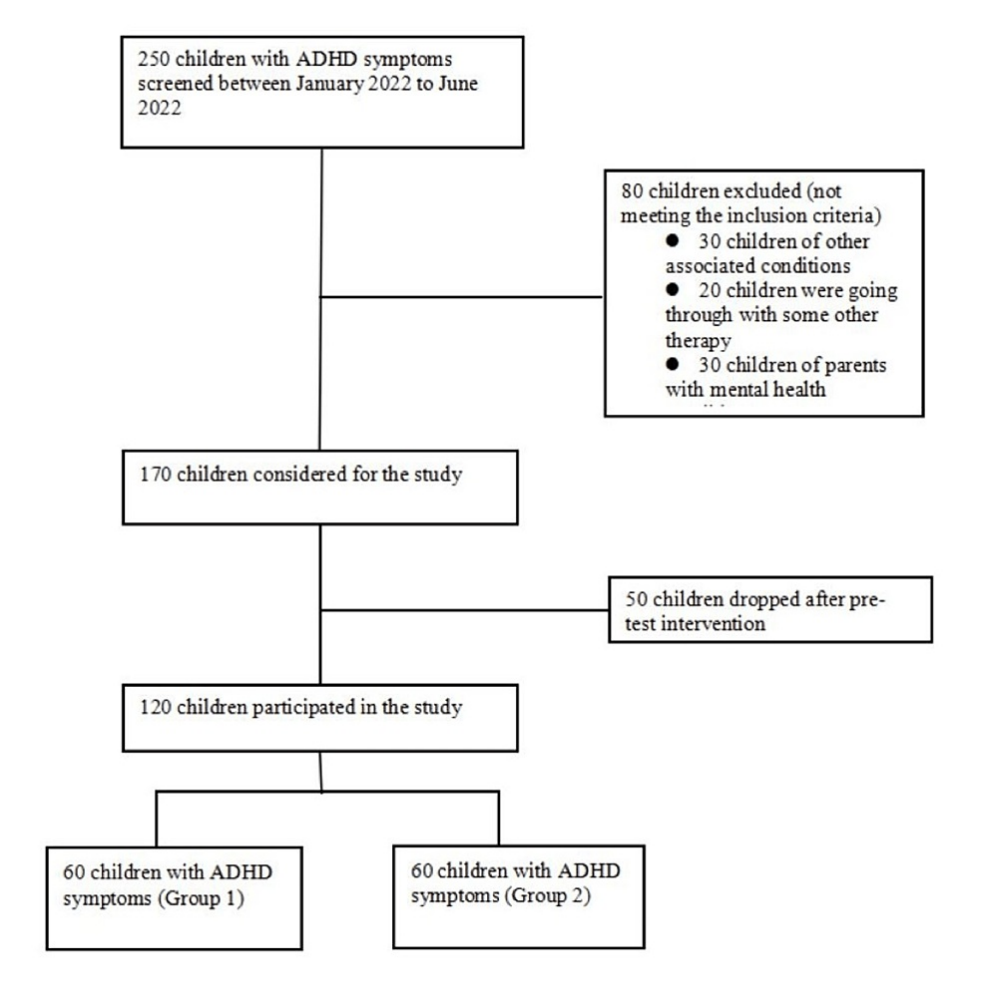
Participants' flow diagram ADHD: attention deficit hyperactivity disorder.

After screening the records using the above-mentioned criteria, a total of 120 children with average intelligence and symptoms of ADHD were included in our study (Figure 1). Out of these, 60 children were part of the intervention group (group 1), and 60 were included in the control group (group 2).

Tools for pre and post-test

Parents and their children completed a demographic questionnaire assessing age, gender, current grade in school, and socio-economic status, along with the education and occupation of their parents. The following tests were used for screening and intervention: ADHD-SRS consisting of 56 items was used to assess the symptoms of ADHD. The normative data are based on age group (5-11 years and 12-18 years), rater (parent and teacher), and gender. A multiple choice test of abstract reasoning, Raven’s Colored Progressive Matrices [9], was used to assess the intelligence level of children. Color Trails 1 and 2 [10] tests were used to assess focused attention in addition to the above functions. In both trails, the subjects were presented with numbers within different colored circles and asked to point out numbers according to the instructions. Digit Span [11] was used for verbal working memory with items presented in forward and reverse conditions with two trails each. Animal Naming Test [12] was used to measure cognitive flexibility by eliciting the words that belong to a particular semantic category. The child was asked to generate the names of as many animals as possible in one minute excluding the names of fish, birds, and snakes. Stroop Color and Word Test [13] was used to assess interference control by presenting the color words in mismatched ink. The test was presented in three levels and the total number of words read correctly and the time taken are considered for final scoring along with an interference score calculated by subtracting the color word score from the color score. The Emotion and Motivation Self-Regulation Questionnaire consists of 36 items answered on a five-point Likert scale that is grouped into learning self-regulation and performance/avoidance self-regulation [14].

The teachers completed the ADHD-SRS rating and the participants who were between the cutoff for the 84th-94th percentile were selected for the study. Permission from the appropriate authorities was obtained before the test was conducted. The parents of these children were then asked for their informed consent and they were informed of the confidentiality of their child's information. Baseline scores were recorded using the above-mentioned tools, followed by post-intervention evaluation after the 60th session using the same tools for comparison.

Intervention procedure

**Figure 2:**
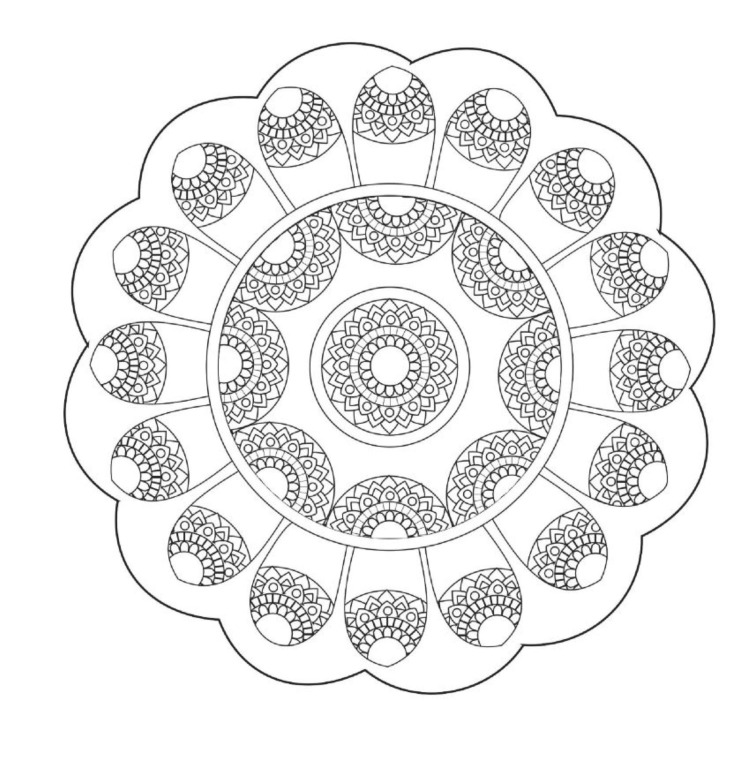
Mandala design used in the study

**Figure 3:**
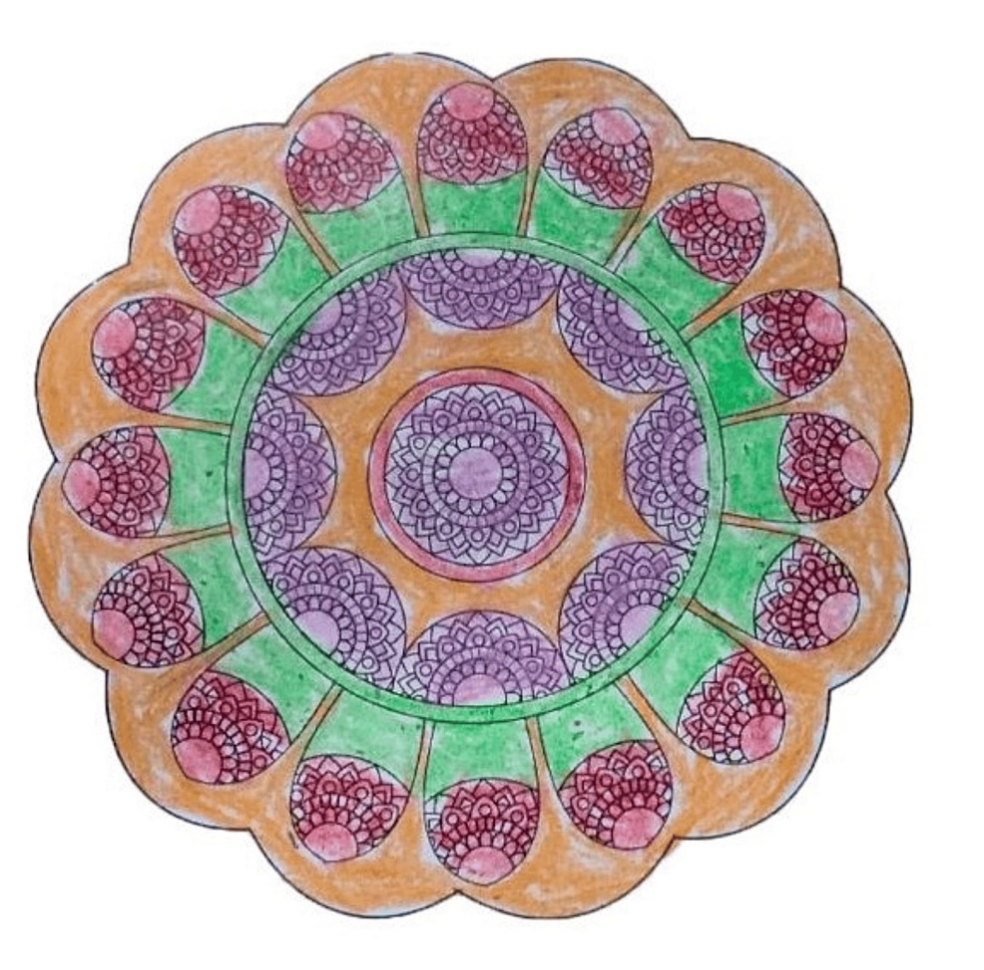
Mandala design colored by a participant

All children used a self-made mandala coloring workbook for intervention under the guidance of researchers and teachers. To develop the workbook, a total of 100 designs were created by a graphic designer. To check the face validity of all these designs, it was administered to 1200 children (each design was colored by 12 students) and the average time taken for coloring was recorded. Based on face validity, the designs that took a similar time for coloring were excluded. Expert validation was sought from five professionals in psychology and art therapy who further shortlisted the designs. Out of the 100 designs, 40 were excluded and the remaining 60 designs were stratified according to the difficulty/time taken for coloring into levels one, two, and three were administered to the child sequentially. An example of mandala design and the coloring done by the participant are shown in Figures 2, 3. The kids in the intervention group received one mandala design from a self-made coloring book every day for up to nine to 10 weeks (a total of 60 sessions).

Statistical analysis

Data were coded and recorded in the Microsoft Excel spreadsheet program (Microsoft Corporation, Redmond, WA). SPSS 23 (IBM Corp., Armonk, NY) was used for data analysis. Descriptive statistics were elaborated in the form of mean/standard deviations. Group comparisons between groups were made using an independent t-test and within the group by using a paired t-test.

## Results

Demographic characteristics and baseline data

The mean age of the children was 8.85 years with 50 boys and 10 girls. The baseline data of demographics, executive functioning, and EMSRQ were comparable between the intervention and control groups. The demographic characteristics and baseline data of the participants are summarized in Table 1.

**Table 1:** Summary of demographic characteristics and baseline data (N = 120)

Demographic data	Intervention group	Control group	p
	n = 60	n = 60	
Age, mean (SD)	9.15 ± 0.88	8.55 ± 1.20	0.006
Male	50 (83.30)	50 (83.30)	1.00
Female	10 (16.70)	10 (16.70)	
Color Trail Test 1	1.95 (0.82)	1.64 (0.78)	0.05
Color Trail Test 2	3.84 (1.38)	3.13 (1.10)	0.00
Digit Span (forward)	5.52 (1.00)	5.78 (1.06)	0.16
Digit Span (backward)	2.20 (0.75)	2.87 (0.83)	0.00
Digit Span (total)	7.73 (1.41)	8.65 (1.39)	0.00
Animal Name Test	8.32 (3.12)	10.57 (3.21)	0.00
Stroop test (W)	73.38 (17.29)	76.97 (11.39)	0.51
Stroop test (C)	61.80 (13.14)	62.23 (12.06)	0.91
Stroop test (CW)	65.60 (16.33)	67.57 (14.64)	0.53
Emotional and motivational self-regulation	65.25 (10.12)	66.35 (12.62)	0.44

Comparison of outcomes between intervention and control groups

The executive functioning of the intervention and control groups was compared (Table 2). There was a significant improvement on Color Trail Test 1 (t = 8.99, p < 0.001), Color Trail Test 2 (t = 8.20, p < 0.001), Digit Span (forward) (t = 7.84, p < 0.001), Digit Span (backward) (t = 2.99, p < 0.05), Digit Span (total) (t = 7.27, p < 0.001), Animal Name Test (t = 7.41, p < 0.001), Stroop words (t = 6.42, p < 0.001), and Stroop color (t = 11.17, p < 0.001). The groups also differed significantly on color-word scores on the Stroop test (t = 7.85, p < 0.001). No significant differences were found between the groups on emotional and motivational self-regulation (t = -1.03, p = 0.29). These results indicate that the participants in the intervention group had significant improvement in areas of focused attention, working memory, cognitive flexibility, and interference control.

**Table 2:** Results between intervention and control group under post-intervention condition

	Intervention	Control	t(118)	p	Cohen's d
	M (SD)	M (SD)			
Color Trail Test 1	0.70 (0.38)	1.68 (0.75)	8.99	<0.001	1.64
Color Trail Test 2	1.76 (0.65)	3.11 (1.10)	8.2	<0.001	1.50
Digit Span (forward)	7.03 (0.58)	5.88 (0.98)	7.84	<0.001	1.43
Digit Span (backward)	3.23 (0.62)	2.85 (0.78)	2.99	<0.05	0.55
Digit Span (total)	10.27 (0.99)	8.73 (1.30)	7.27	<0.001	1.33
Animal Name Test	16.37 (3.76)	11.68 (3.14)	7.41	<0.001	1.35
Stroop test (word)	91.65 (12.19)	78.15 (10.81)	6.42	<0.001	1.17
Stroop test (color)	84.35 (9.47)	63.10 (11.29)	11.17	<0.001	2.04
Stroop test (color-word)	87.25 (11.71)	68.52 (14.30)	7.85	<0.001	1.43
Emotional and motivational self-regulation	64.73 (11.77)	67.00 (12.34)	1.03	0.20	0.19

Comparison of outcomes between the pre and post-test of intervention groups

The executive functioning of the intervention group in pre and post-intervention was compared. The paired t-test was applied to mean scores of the various measures of executive functioning. Table 3 represents significant difference on Color Trail Test 1 (t = 13.64, p < 0.001), Color Trail Test 2 (t = 14.78, p < 0.001), Digit Span (forward) (t = 12.40, p < 0.001), Digit Span (backward) (t = 9.98, p = 0.003), Digit Span (total) (t = 13.82, p < 0.001), Animal Name Test (t = 19.07, p < 0.001), Stroop words (t = 12.09, p < 0.001), and Stroop color (t = 17.79, p < 0.001). The groups also differed on color-word scores on the Stroop test (t = 16.24, p < 0.001). No significant differences were found in emotional and motivational self-regulation between pre and post-tests (t = 0.29, p = 0.72).

**Table 3:** Results between pre and post-intervention for intervention group participants

	Pre	Post	t(59)	p	Cohen's d
	M (SD)	M (SD)			
Color Trail Test 1	1.95 (0.82)	0.70 (0.38)	13.64	<0.001	1.76
Color Trail Test 2	3.84 (1.38)	1.76 (0.65)	14.78	<0.001	1.91
Digit Span (forward)	5.52 (1.00)	7.03 (0.58)	12.4	<0.001	1.60
Digit Span (backward)	2.20 (0.75)	3.23 (0.62)	9.98	<0.001	1.29
Digit Span (total)	7.73 (1.41)	10.27 (0.99)	13.82	<0.001	1.78
Animal Name Test	8.32 (3.12)	16.37 (3.76)	19.07	<0.001	2.46
Stroop Test (word)	73.38 (17.29)	91.65 (12.19)	12.09	<0.001	1.56
Stroop Test (color)	61.8 (13.14)	84.35 (9.47)	17.79	<0.001	2.30
Stroop Test (color-word)	65.60 (16.33)	87.25 (11.71)	16.24	<0.001	2.10
Emotional and motivational self-regulation	65.25 (10.12)	64.73 (11.77)	0.29	0.72	0.04

There was no significant difference observed between the pre and post-test results of the control group. However, significant improvement was found in the intervention group (Table 4).

**Table 4:** Results between pre and post-intervention for control group participants

	Pre	Post	t(59)	p	Cohen's d
	M (SD)	M (SD)			
Color Trail Test 1	1.64 (0.78)	1.68 (1.10)	2.54	0.01	0.33
Color Trail Test 2	3.13 (1.10)	3.11 (1.10)	0.56	0.68	0.07
Digit Span (forward)	5.78 (1.06)	5.83 (1.03)	1.76	0.08	0.23
Digit Span (backward)	2.87 (0.83)	2.85 (0.78)	0.57	0.77	0.07
Digit Span (total)	8.65 (1.39)	8.73 (1.30)	1.69	0.11	0.22
Animal Name Test	10.57 (3.21)	10.83 (3.18)	1.78	0.08	0.23
Stroop Test (word)	76.83 (11.18)	77.25 (10.72)	1.67	0.10	0.22
Stroop Test (color)	62.23 (12.06)	62.62 (11.64)	1.72	0.09	0.22
Stroop Test (color-word)	67.57 (14.64)	67.85 (14.49)	1.96	0.06	0.25
Emotional and motivational self-regulation	66.35 (12.62)	66.62 (12.42)	1.85	0.07	0.24

These results indicate that attention span was the parameter that showed the maximum improvement with the intervention of mandala coloring. Working memory, cognitive flexibility, and interference control also showed statistically significant improvement with the intervention. After 60 sessions of intervention in the follow-up session, parents also reported improvement in academic performance and attention.

## Discussion

The study examined the efficacy of mandala coloring intervention on executive functioning and emotional and motivational self-regulation of children with symptoms of ADHD. The study shows that mandala coloring intervention may effectively increase the executive functioning of children with symptoms of ADHD. These results correspond with the research findings of Auerbach & Richardson and Kaimal et al. that indicate that a coloring program that incorporated mandala coloring helped children and adolescents with ADHD symptoms and executive function issues [15,16]. As a relaxing and stress-relieving activity, coloring can also help children gain better control of their emotions, focus, and behavior. Coloring mandalas can work as a kind of mindfulness meditation, helping kids focus on the present moment and lessen distractions, leading to a positive impact on attention in children with ADHD symptoms. Multiple studies have reported an improvement in the attention span of children who engage in coloring activities [17].

There were significant differences between the intervention and control groups in terms of working memory. Mandala coloring is a well-liked art therapy technique that has been claimed to encourage relaxation and enhance focus. Similarly, the ability to adapt to situations, decision-making ability, social skills, and self-control have been increased and the children were able to adjust themselves to new situations that represent significant improvement between intervention and control groups in terms of cognitive flexibility.

Wahbeh et al. suggested the positive effect of mandala coloring on interference control in a group of people with ADHD symptoms [18]. The current study found that there was an improvement in cognitive control of children that involves selectively attending to relevant information while inhibiting irrelevant responses, and coloring mandalas has a beneficial impact on interference control in children with ADHD symptoms.

Mandala coloring intervention is proven to be effective for children with symptoms of ADHD in areas of executive functioning; however, its effectiveness is limited in the area of EMSRQ. Some children may have specific needs that mandala coloring intervention as a standalone modality does not address adequately. The affecting external factors are family issues or other prominent ongoing challenges. Few children in the intervention group did not show significant improvement. On further evaluation, it was found that they had inconsistent routines and excessive distractions as their parents had personal conflicts, and maintaining a conducive environment for the study was not possible. Some children were not exhibiting interest during the intervention and hence the chosen intervention may not be suitable for their specific needs.

Mandala coloring is easy to use because its simple designs require minimal artistic skills and promote relaxation and focus. It can be used at home by parents without any supervision. It is suitable for people of all ages from children to adults. This intervention can be used as an educational tool to teach children about different shapes, patterns, and colors. It will also be effective for children with other behavioral disorders.

However, the observed limitations of the study include a relatively small sample size, non-random allocation of the participants in the intervention group, and lack of long-term follow-up. Hence, further studies on this subject are needed to ascertain whether these improvements are sustained over a longer period of time. A larger sample size with longer follow-ups may provide a better understanding. Using an equal ratio of participants in terms of gender could help as an additional variable. We intend to carry out a future randomized trail with a longer follow-up as part of our ongoing investigation into this topic.

## Conclusions

The current study indicates that mandala coloring intervention can improve executive functioning in children; however, its effectiveness is limited in addressing EMSRQ. Mandala coloring may help children and adolescents perform better academically, socially, and in daily life activities by improving working memory, cognitive flexibility, attention, and interference control. Further studies with a larger sample size need to be undertaken to unravel the potential of this simple yet effective intervention.

## References

[1] Xie GH, Wang Q (2021). Mandala coloring as a therapeutic tool in treating stress-anxiety-depression syndrome. Asian J Interdiscip Res.

[2] Jung CG (2017). Mandala Symbolism. https://press.princeton.edu/books/hardcover/9780691654614/mandala-symbolism.

[3] Cox CT, Cohen BM (2000). Mandala artwork by clients with DID: clinical observations based on two theoretical models. Art Ther.

[4] Smitherman-Brown V, Church RP (1996). Mandala drawing: facilitating creative growth in children with ADD or ADHD. Art Ther.

[5] Couch JB (1997). Behind the veil: mandala drawings by dementia patients. Art Ther.

[6] Epperson J, Valum J (1992). The effects of stimulant medications on the art products of ADHD children. Art Ther.

[7] Curry NA, Kasser T (2005). Can coloring mandalas reduce anxiety?. Art Ther.

[8] Babouchkina A, Robbins SJ (2015). Reducing negative mood through mandala creation: a randomized controlled trial. Art Ther.

[9] Raven Raven, J. C. (1936) (1936). Raven JC. The performances of related individuals in tests mainly educative and mainly reproductive mental tests used in genetic studies. https://www.worldcat.org/title/1006059359.

[10] D’Elia LF, Satz P, Uchiyama CL, White T (1996). Color Trails Test. https://www.parinc.com/Products/Pkey/77.

[11] Wechsler D (1997). Adult Intelligence Scale. https://psycnet.apa.org/doiLanding?doi=10.1037%2Ft49755-000.

[12] Lezak M (1995). Neuropsychological Assessment. https://www.google.co.in/books/edition/Neuropsychological_Assessment/kj-RO9NyCgkC?hl=en.

[13] Golden CJ (1978). Stroop Color and Word Test: A Manual for Clinical and Experimental Uses. https://www.scirp.org/(S(lz5mqp453edsnp55rrgjct55.))/reference/referencespapers.aspx?referenceid=1178750.

[14] Alonso-Tapia J, Panadero Calderón E, Díaz Ruiz MA (2014). Development and validity of the emotion and motivation self-regulation questionnaire (EMSR-Q). Span J Psychol.

[15] Auerbach JG, Richardson PA (2007). Treatment of childhood ADHD with mindfulness meditation: a case study. J Child Fam Stud.

[16] Kaimal G, Ray K, Muniz J (2016). Reduction of cortisol levels and participants’ responses following art making. Art Ther (Alex).

[17] Semple RJ, Droutman V, Reid BA (2017). Mindfulness goes to school: things learned (so far) from research and real-world experiences. Psychol Sch.

[18] Wahbeh H, Goodrich E, Goy E (2020). Feasibility and outcomes of an art therapy-based intervention for adults with attention-deficit/hyperactivity disorder. Art Ther.

